# Holding a wing horizontal: Roles for muscles of the pectoral girdle other than the main two flight muscles

**DOI:** 10.1111/joa.70051

**Published:** 2025-09-25

**Authors:** D. Charles Deeming, María Clelia Mosto

**Affiliations:** ^1^ Joseph Banks Laboratories, School of Natural Sciences University of Lincoln Lincoln UK; ^2^ División Zoología Vertebrados, Museo de La Plata‐Facultad de Ciencias Naturales y Museo Universidad Nacional de La Plata – CONICET. Buenos Aires Argentina

**Keywords:** allometry, birds, horizontal wing, humerus, *m. Deltoideus major*, *m. Pectoralis*, *m. Scapulohumeralis caudalis*, *m. Supracoracoideus*, soaring flight

## Abstract

Whilst many birds glide briefly with wings held horizontally, some species maintain this posture for extended periods during soaring. This is considered possible because of the contraction of the *m. pectoralis* that holds the wing in place, although albatrosses seem to have a physical shoulder lock that helps with this action. However, studies of this flight style have not considered the cranially orientated long‐axis rotation of the humerus induced by the contraction of the main flight muscles that would depress the ulna and change the angle of the aerofoil downwards. This study explored whether the *m. deltoideus major* helps counteract this rotation. Muscle masses were collated from the literature and from dissections of birds to allow exploration of the allometry of muscle masses versus body mass. All muscles exhibited isometry with body mass, but relative to the size of the *m. pectoralis*, the *m. deltoideus major* was large but only in a few species that regularly soar or glide for long periods. By contrast, other elevator muscles were less variable among species. The presence of relatively large *deltoideus major* muscles in soaring species was suggestive that this muscle, since it originates on the scapula extending caudally and inserting on the dorsal humerus, may counteract humeral long‐axis rotation around its longitudinal axis during contraction of the breast muscles. The results of this study are suggestive of previously unconsidered substantial roles for other muscles of the pectoral girdle and forelimb during different flight styles in birds.

## Graphical abstract

This report explores which muscles of the pectoral girdle are employed to allow birds to hold their wings horizontally with a level aerofoil surface during a glide.

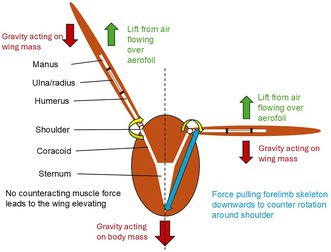

## 1 INTRODUCTION

**FIGURE 1:**
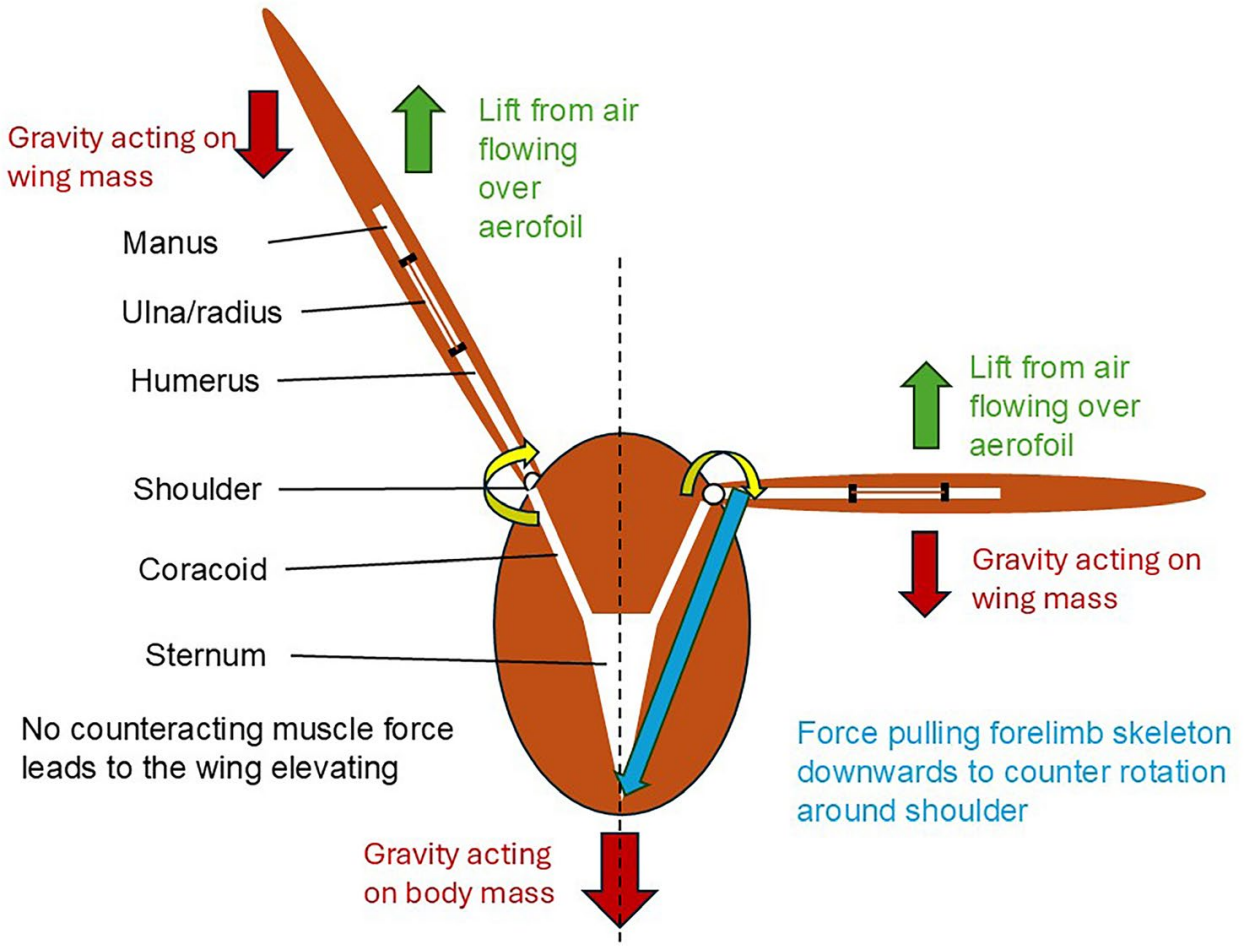
Diagrammatic representation of the wing from the cranial aspect when the specimen is in the air. The left‐hand side indicates, in the absence of a force resisting dorso‐ventral long‐axis rotation around the shoulder, the tendency of the wing to elevate caused by gravity pulling the body downwards and lift pulling the wing upwards. The right‐hand side indicates how the force applied by the *m. pectoralis* pulls the humerus down, resisting the lift generated by the aerofoil so that the wing can be held horizontally.

A key feature of flight in birds is that they hold their wings horizontally during gliding or soaring flight (Meyers, 1997; Meyers & Mathias, 1997; Meyers & McFarland, 2016; Meyers & Stakebake, 2005). The shoulder is a multiaxial joint that has no physical restriction for elevation/depression, movement cranially/caudally, and the humerus can rotate around its longitudinal axis (Rayner, 1988). Hence, if the extended wing is to be effective as an aerofoil in forward flight, there has to be a morphological feature that counters the downward force of gravity on the body and upward lift on the wing, both of which will make the wing elevate (Figure 1). Pennycuick (1972) stated that there has to be a steady force exerted downwards by the *m. pectoralis* to hold the wing horizontal (Figure 1), and electromyographic studies of birds have shown that the *m. pectoralis* is active during gliding flight (Goldspink et al., 1978; Meyers, 1993; Tobalske & Dial, 1994).

Moreover, Pennycuick (1982) suggested that, in larger petrels and albatrosses (Procellariiformes), the contractile action of the *m. pectoralis* was supplemented by a ‘shoulder lock’ that prevented elevation of the humerus above horizontal during gliding. This lock consisted of a fan‐shaped, tendinous structure positioned deep to the belly of the superficial *m. pectoralis* with a long origin along the keel of the sternum and an insertion on the deltopectoral crest of the dorsal humerus. In dead birds, this tendon prevented the wing from elevating until it was cut and was considered to reduce the energetics of the muscle during long‐term gliding flight (Pennycuick, 1982). By contrast, Meyers and Stakebake (2005) reported that the tendon was deep within a separated ‘deep’ belly of the *m. pectoralis* in albatrosses, but it did also insert on the deltopectoral crest cranially to the superficial *m. pectoralis*, although there was also an insertion on to the biceps tendon. Divided pectoralis muscles seem to be relatively common in various large birds that regularly soar (see Meyers & Mathias, 1997).

Another aspect of muscle action during gliding involves the types of muscle fibres involved in contraction. Double‐crested cormorants (*Nannopterum auritum*) lack waterproofed feathers and engage in a wing drying posture, which, although not gliding flight, involves holding the wings extended for a prolonged period of time. Meyers (1997) suggested that this behaviour was possible because of numerous slow‐twitch, or slow‐tonic, muscle fibres in several forelimb and pectoral girdle muscles associated with wing extension. Slow muscle fibres also seemed to be involved in the maintenance of gliding in albatrosses (Meyers & Stakebake, 2005) but not in species like the California Gull (*Larus californicus*), which also lack a divided *m. pectoralis* (Meyers & Mathias, 1997). Similarly, there are other birds, for example, the American Kestrel (*Falco sparverius*), that do not seem to have any anatomical specialisations for gliding (Meyers, 1992).

Such studies suggest that holding an outstretched wing horizontal is achieved by contraction of the *m. pectoralis*, combined, in some species of larger petrels and albatrosses, by a tendinous ‘lock’ that limits elevation of the humerus (Meyers & Stakebake, 2005; Pennycuick, 1982). A scapular anchor was observed in the American Kestrel (*Falco sparverius*) and other species (Canova et al., 2015; Meyers, 1993; Razmadze et al., 2018). This band of connective tissue originates on the scapula and inserts onto the *m. deltoideus major* and may play a role as a mechanical stop to limit protraction of the humerus or be involved in sensing the position of the shoulder joint (Meyers, 1993).

However, a key element of deploying the *m. pectoralis* to hold a wing horizontally is the fact that the *m. pectoralis* is located cranially to the humerus and inserts on the deltopectoral crest located on the dorsal aspect of the humerus. Hence, as the *m. pectoralis* contracts to depress the humerus, such contraction will pull the point of insertion forward, causing the humerus to rotate around its long axis in a cranial direction at the shoulder. This long‐axis rotation will also rotate the distal extremity of the humerus at the elbow and depress the ulna/radius, which will tilt the aerofoil surface dorsally and slightly increase the gliding angle, making the bird descend faster (Norberg, 1990). Observations of gliding birds show, however, that the plane of the wing is notably near to horizontal during gliding. It seems logical that the *m. pectoralis* is involved in holding a wing horizontally, but the effects of long‐axis rotation of the humerus do not seem to have been considered to date.

The question arises as to how horizontal gliding is possible if contraction of the *m. pectoralis* rotates the humerus? Given that in flapping flight the humerus is elevated during an upstroke by the *m. supracoracoideus*, perhaps this muscle contracts to counter the downstroke of the *m. pectoralis*, thus allowing the wing to be held horizontal during gliding. However, in most birds, the tendon extending from the *m. supracoracoideus* and passing through the trioseal canal inserts cranially on the dorsal aspect of the humerus more proximal than the deltopectoral crest (Heers et al., 2018; Lo Coco et al., 2020; Meyers, 1992; Picasso & Mosto, 2018; Proctor & Lynch, 1993). Contraction of the *m. supracoracoideus* to effect elevation of the humerus would, therefore, also pull the deltopectoral crest cranially and rotate the humerus through its long axis in that direction (Poore et al., 1997).

Logically, there should be a muscle in the pectoral girdle that inserts dorsally on the proximal humerus and originates caudally, which, upon contraction, rotates the humerus caudally around the plane of articulation at the shoulder. Moreover, this muscle should be relatively large in order to counter the contraction of the large *m. pectoralis*, which comprises, on average, 16% of the body mass of the bird (Deeming, 2023). There are numerous muscles involved in the positioning and movement of the wing relative to the pectoral girdle (Baumel et al., 1979; Proctor & Lynch, 1993), but relatively few muscles insert on the proximal humerus, extend caudally, and are large. The *m. deltoideus major* fits these criteria because it originates on the scapula, which is caudal to the shoulder joint, and inserts on the dorsal side of the proximal humerus (Heers et al., 2018; Lo Coco et al., 2020; Picasso & Mosto, 2018). Contraction of the *m. deltoideus major* will elevate the humerus, but it will rotate caudally around its longitudinal axis (Dial et al., 1991). The *m. scapulohumeralis caudalis* is also a muscle of similar size to the *m. deltoideus major* in many bird species that originates on the scapula but inserts onto the ventral aspect of the proximal humerus. Hence, contraction of the *m. scapulohumeralis caudalis* will elevate the humerus whilst rotating the bone through its long axis cranially. The *m. deltoideus major* is, therefore, a candidate to counter the cranially orientated long‐axis rotation caused by the contraction of the *m. pectoralis* as it depresses the humerus to hold it horizontal by resisting the downward pull of gravity on the body and the upward pull of lift on the wing (Figure 1).

Flight style of a species may also be important in determining whether the long‐axis rotation of the humerus has to be countered. Species like raptors and large birds that soar tend to not flap their wings and so will need to hold their wings horizontally for extended periods of time. Many of these birds have high mass ratios between the *m. pectoralis* and *m. supracoracoideus*, indicating relatively weak upstrokes (Deeming, 2023). It can be predicted that birds that regularly glide or soar will have a relatively large *m. deltoideus major* needed to counter the *m. pectoralis*. By contrast, other species that rely on near‐continuous flapping, and so do not hold their wings horizontally for extended periods of time, will not need such a large *m. deltoideus major*. Therefore, the ratio of the masses of the *m. pectoralis* and *m. deltoideus major* will be variable among orders, being smaller in gliding birds compared with flapping birds. By contrast, given that the *m. scapulohumeralis caudalis* will not be involved in countering the long‐axis rotation of the humerus, its mass ratio relative to the *m. pectoralis* will be less variable.

This study explored the inter‐relationships between body mass and the masses of the *m. pectoralis* and three muscles involved in elevating the humerus, that is, the *m. supracoracoideus*, *m. deltoideus major*, and *m. scapulohumeralis caudalis*. Data were collated from the literature and from previously unreported studies of the musculature of the pectoral girdle to test the following hypotheses. (1) There would be isometric relationships between body mass and the mass of each of the muscles. (2) Given previous results for breast muscles (Deeming, 2023), order would have a significant effect on muscle masses. (3) Variability in the data for the ratio of the mass of the *m. pectoralis* to the mass of each of the elevator muscles would vary, with values for the *m. deltoideus major* exhibiting the greatest variation. (4) In those orders that rely on gliding or soaring flight, for example, the Accipitriformes, the *m. deltoideus major* muscle would be significantly larger compared with values predicted based on values standardised against the mass of the *m. pectoralis*.

## 2 METHODS

Values for the masses (in grams) of the *m. pectoralis* (P), *m. supracoracoideus* (SC), and *m. scapulohumeralis caudalis* (SHC) were collated from unpublished bird dissection studies conducted by MCM and from the literature (Bribiesca‐Contreras et al., 2019, 2021; Calmaestra & Moreno, 2005; Hedrick et al., 2004; Heers et al., 2018; Mosto et al., 2022; Picasso & Mosto, 2018; Razmadze et al., 2018; Yang et al., 2015). Data for the mass of the *m. deltoideus major* (g; DM) were recorded; whenever the *m. deltoideus minor* was larger, its mass was recorded instead. It was noted that for muscle mass data for the *m. pectoralis*, *m. supracoracoideus*, and *m. deltoideus major* for passerine species reported by Calmaestra and Moreno (2005) were considered to be very low. When data for the mass of the *m. pectoralis* were compared with that reported by Deeming (2023), the values published by Calmaestra and Moreno (2005) were an order of magnitude smaller. Therefore, prior to use in the current analysis, data published by Calmaestra and Moreno (2005) were multiplied by 10 to correct this difference. If reported, body mass (g) was recorded for the species, but if the body mass was not available, then mean species values from Dunning (2008) were used. If there were multiple reports for a species, then these were averaged prior to analysis. Details of the 97 species, representing 18 orders, for which mean mass values were available for the *m. pectoralis*, *m. supracoracoideus*, and *m. deltoideus major*, are given in Table S1 of the supplementary materials. Data for the mass of the *m. scapulohumeralis caudalis* were unavailable for the passerine species included in Calmaestra and Moreno (2005), as well as for a few other species (Table S1). As a result, the final dataset for the analysis of the data for the *m. scapulohumeralis caudalis* comprised only 75 species representing 18 orders (Table S1).

All data were log_10_‐transformed prior to phylogenetically controlled generalised linear modelling (pglm) performed in R version 4.2.3 (R Core Development Team, 2024) as described previously (Deeming, 2023; Deeming et al., 2022, 2024, 2025). The analysis used code provided by Carl Soulsbury (pers. comm.) and required the R packages *ape* (Paradis & Schliep, 2019), *mvtnorm* (Genz et al., 2021), and *MASS* (Venables & Ripley, 2002). A phylogenetic tree of the species in the dataset was produced by pruning a tree of all bird species based on the Hackett backbone using vertlife.org (Jetz et al., 2014). The analysis calculated a phylogenetic signal (*λ*), where *λ* = 0 indicates no phylogenetic signal in the residuals and suggests that trait variation is independent of shared ancestry. If *λ* = 1 the observed covariance in residuals matches the expectations under a Brownian motion model of trait evolution (Freckleton et al., 2002).

Initial analyses tested whether the phylogenetically controlled relationships between body mass and the four muscle masses conformed to isometry. Second, masses of the three elevator muscles were tested against the mass of the *m. pectoralis* as an independent variable. Similarly, masses of the *m. deltoideus major* and *m. scapulohumeralis caudalis* were tested against the mass of the *m. supracoracoideus*. Finally, the allometric relationship between the masses of the *m. deltoideus major* and *m. scapulohumeralis caudalis* was explored. In each case, slopes of regression analyses were compared against an isometric relationship with a slope of one using a one‐sample, two‐tailed *t*‐test (Bailey, 1981).

Previous analysis of allometry of breast muscle masses demonstrated a significant effect of order (Deeming, 2023). This was explored here using a smaller dataset by excluding any orders represented by fewer than five species, leaving 69 species across six avian orders. For the *m. scapulohumeralis caudalis*, data reduced the sample size to 45 species from five orders. Analysis involved pglm to test for the effect of order as a fixed factor and log body mass as a covariate. An interaction term was included in the initial model, but if it was non‐significant, then the model was revised to exclude the interaction term and was re‐run. This analysis was phylogenetically controlled as wide variation in body mass within an order that could have biased the results (Deeming & Ferrari Da Silva, 2025).

To control for body size in comparisons across species and orders, the mass of the *m. pectoralis* was divided by the mass of each of the three elevator muscles. A high ratio value indicated that the muscle in question was small relative to the mass of the *m. pectoralis*. Variance ratio tests (i.e., highest variance/lowest variance) were used to compare variance values calculated for the full dataset for each muscle to assess whether differences in variation between muscle groups were statistically significant.

Residuals for each species were calculated for each of the three elevator muscles as the difference between the observed muscle mass and the mass predicted by the phylogenetically controlled regression on *m. pectoralis* mass. These residuals were then standardised by expressing them as a percentage of the actual muscle mass. Order means of these standardised residuals were tested against a mean of zero using a one‐sample *t*‐test, but only for orders represented by at least three species in the dataset.

## 3 RESULTS

Summary statistics for the mean mass of the body and each of the four pectoral girdle muscle masses are given in Table 1. In general, larger muscle masses were associated with larger birds, with the *m. pectoralis* being the heaviest muscle recorded. In some orders, for example, the Accipitriformes, Falconiformes, and Piciformes, the masses of the three elevator muscles were comparable in size. In most other orders, for example, Charadriiformes, Columbiformes, or Gaviiformes, the *m. supracoracoideus* was much heavier than the other two elevator muscles, which were often of similar sizes (Table 1).

**TABLE 1:** Summary statistics for each order in terms of species represented, body mass, and the masses of pectoral girdle muscles.

	*N*	Body mass (g)	*m. Pectoralis* mass (g)	*m. Supracoracoideus* mass (g)	*m. Deltoideus* major/minor mass (g)	*m. Scapulohumeralis caudalis* mass (g)
Accipitriformes^a^	18	2298 ± 662 [238–11,500]	129.5 ± 28.8 [13.9–412.0]	8.69 ± 3.09 [0.71–57.0]	9.99 ± 2.74 [1.18–42.0]	7.36 ± 1.81 [0.92–30.00]
Anseriformes	4	2319 ± 789 [870–4400]	191.5 ± 69.8 [75.5–390.0]	19.88 ± 5.18 [9.37–33.37]	6.10 ± 2.34 [2.26–12.53]	11.96 ± 4.18 [4.06–22.45]
Charadriiformes	7	431 ± 114 [97–822]	25.61 ± 7.30 [5.31–47.06]	4.52 ± 2.08 [0.95–16.68]	0.65 ± 0.18 [0.19–1.23]	1.34 ± 0.37 [0.34–2.65]
Columbiformes	3	254 ± 62.2 [136–347]	33.05 ± 9.19 [15.8–47.19]	5.80 ± 1.35 [3.15–7.53]	0.68 ± 0.21 [0.32–1.03]	1.68 ± 0.50 [0.71–3.32]
Falconiformes	10	386 ± 123 [104–347]	25.83 ± 9.79 [4.1–94.01]	1.63 ± 0.64 [0.35–5.59]	2.15 ± 0.89 [0.26–7.59]	1.17 ± 0.41 [0.36–4.53]
Galliformes	3	1007 ± 385 [541–1770]	36.01 ± 3.59 [28.83–39.73]	10.01 ± 1.44 [8.49–12.89]	1.43 ± 0.60 [0.72–2.63]	3.52 ± 0.39 [2.77–4.08]
Gaviiformes	1	1255	47.76	4.28	0.869	1.387
Gruiiformes	1	737	38.93	6.85	2.045	2.835
Passeriformes^b^	22	48 ± 16 [7–357]	6.41 ± 1.34 [1.09–25.62]	0.65 ± 0.12 [0.161–2.110]	0.228 ± 0.062 [0.042–1.400]	1.39 [0.58–2.20]
Pelecaniformes	2	2068 [935–3200]	107.9 [94.8–120.9]	10.33 [8.24–12.42]	4.44 [3.39–5.48]	5.11 [3.54–6.67]
Piciformes	6	298 ± 78 [140–618]	15.24 ± 4.46 [6.98–37.0]	1.61 ± 0.48 [0.78–3.97]	1.35 ± 0.40 [0.86–3.33]	1.28 ± 0.42 [0.69–3.34]
Podicipediformes	2	1035 [424–1646]	16.2 [5.6–26.9]	2.19 [0.86–3.52]	0.575 [0.16–0.99]	0.585 [0.22–0.95]
Procellariiformes	5	409 ± 111 [159–761]	14.84 ± 5.01 [5.13–33.47]	1.76 ± 0.49 [0.58–3.04]	0.41 ± 0.12 [0.13–0.83]	0.69 ± 0.22 [0.29–1.53]
Psittaciformes^c^	4	230 ± 52 [87.3–333]	12.86 ± 1.89 [9.1–18.0]	2.30 ± 0.39 [1.41–3.30]	0.39 ± 0.16 [0.15–0.81]	0.71 ± 0.15 [0.51–1.00]
Sphenisciformes	3	3200 ± 513 [2500–4200]	138.10 ± 22.30 [93.8–164.6]	62.37 ± 9.19 [44.02–72.53]	2.65 ± 0.31 [2.4–3.27]	12.82 ± 0.60 [11.43–13.35]
Strigiformes	3	163 ± 84 [77.2–330]	8.88 ± 5.09 [3.42–19.06]	0.57 ± 0.34 [0.21–1.24]	0.27 ± 0.05 [0.18–0.32]	0.52 ± 0.30 [0.21–1.11]
Suliformes	2	1979 [1034–2924]	119 [43.5–194.6]	8.93 [6.16–11.7]	3.21 [2.15–4.27]	4.31 [2.52–6.1]
Tinamiformes	1	260	20.07	6.41	0.31	0.38

*Note*: Values are mean ± SE with a range of values in brackets. SE values are only presented if *N* ≥ 3.

^a^ *N* = 17.

^b^ *N* = 2.

^c^ *N* = 3 in orders indicated for data for *m. scapulohumeralis caudalis* mass.

**FIGURE 2:**
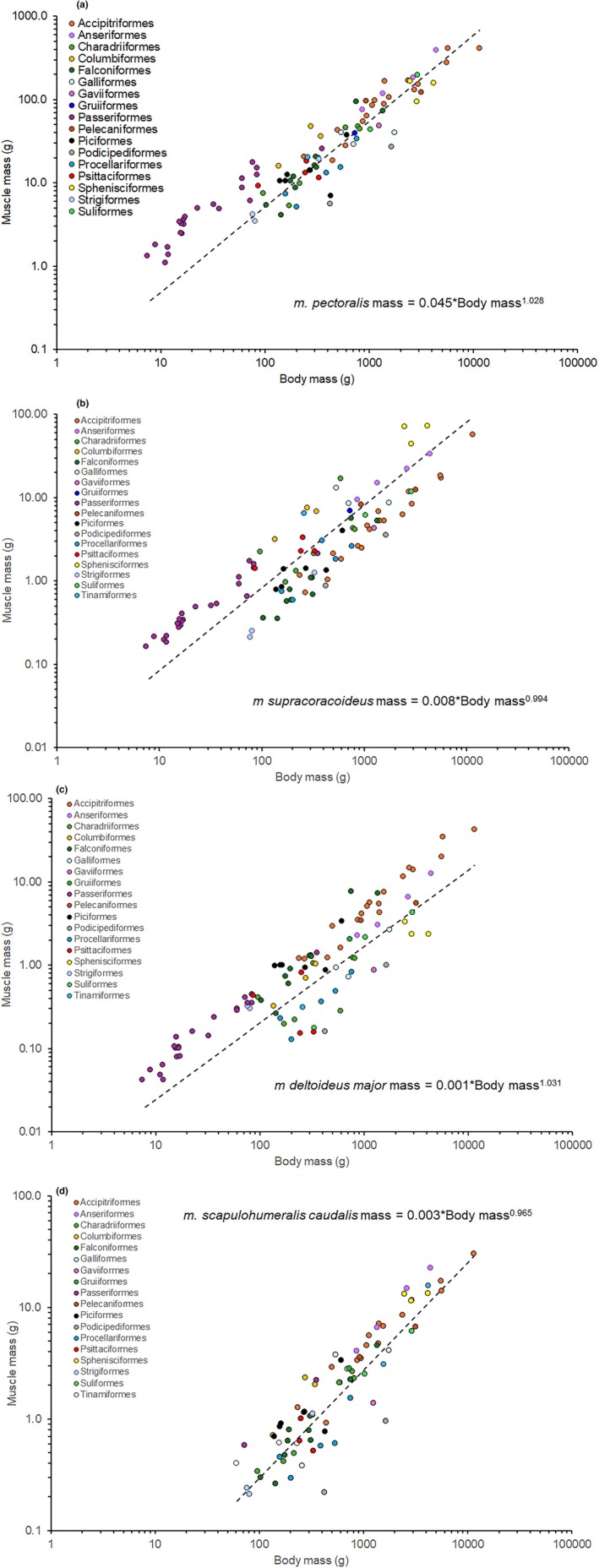
Relationships between body mass and the mass of (a) the *m. pectoralis* (top left panel), (b) *m. supracoracoideus* (top right panel), (c) *m. deltoideus major* (bottom left panel), and (d) *m. scapulohumeralis caudalis* (bottom right panel). Note the log–log axes. The dashed lines indicate phylogenetically controlled regression estimates through all of the data points as indicated.

All four muscles studied here exhibited highly significant positive relationships with body mass, and none of the slopes of the regression estimates significantly differed from an isometric slope of 1.0 (Table 2; Figure 2). When the three smaller elevator muscles were compared with the mass of the *m. pectoralis*, the *m. supracoracoideus* and *m. deltoideus major* both exhibited isometry, but the m. *scapulohumeralis caudalis* exhibited significant negative allometry (Table 2). The *m. deltoideus major* exhibited isometry with the *m. supracoracoideus*, but the *m*. *scapulohumeralis caudalis* exhibited significant negative allometry with both muscles (Table 2). Each of the relationships had a high phylogenetic signal (>0.75; Table 2), and the distribution of points for orders with multiple species suggested that at least, some of these orders would have different intercepts from those of these general models (Figure 2).

**TABLE 2:** Results of phylogenetically controlled regression analysis of the relationships between body mass and the masses of the four muscles of interest in the pectoral girdle.

	Slope (SE)	Intercept (SE)	Slope *t*‐value (*p*‐value)	Intercept *t*‐value (*p*‐value)	Model *F*‐value^a^ (*p*‐value)	*R* ^2^	*λ*	Departure from isometry: *t*‐value (*p*‐value)
Body mass								
*m. Pectoralis*	1.028 (0.052)	−1.343 (0.223)	19.79 (<0.0001)	−6.03 (<0.0001)	391.79 (<0.0001)	0.805	0.853	0.54 (0.592)
*m. Supracoracoideus*	0.994 (0.058)	−2.072 (0.324)	17.07 (<0.0001)	−6.39 (<0.0001)	291.51 (<0.0001)	0.754	0.959	−0.10 (0.918)
*m. Deltoideus*	1.031 (0.077)	−2.890 (0.328)	13.45 (<0.0001)	−8.80 (<0.0001)	180.93 (<0.0001)	0.656	0.852	0.40 (0.688)
*m. Scapulohumeralis caudalis*	0.965 (0.053)	−2.460 (0.250)	18.37 (<0.0001)	−9.83 (<0.0001)	337.41 (<0.0001)	0.822	0.936	−0.69 (0.491)
*m. Pectoralis*								
*m. Supracoracoideus*	0.945 (0.036)	−0.744 (0.227)	25.94 (<0.0001)	−3.27 (<0.0001)	673.07 (<0.0001)	0.876	0.973	−1.52 (0.130)
*m. Deltoideus*	0.976 (0.053)	−1.500 (0.188)	18.43 (<0.0001)	−7.99 (<0.0001)	339.82 (<0.0001)	0.782	0.758	−0.45 (0.652)
*m. Scapulohumeralis caudalis*	0.936 (0.031)	−1.226 (0.151)	29.74 (<0.0001)	−8.10 (<0.0001)	884.52 (<0.0001)	0.924	0.949	**−2.06 (0.043)**
*m. Supracoracoideus*								
*m. Deltoideus*	0.879 (0.072)	−0.645 (0.428)	12.24 (<0.0001)	−1.51 (0.131)	149.91 (<0.0001)	0.612	0.968	−1.68 (0.096)
*m. Scapulohumeralis caudalis*	0.888 (0.041)	−0.422 (0.179)	21.53 (<0.0001)	−2.36 (0.021)	463.60 (<0.0001)	0.864	0.932	**−2.73 (0.008)**
*m. Deltoideus*								
*m. Scapulohumeralis caudalis*	0.716 (0.059)	0.196 (0.286)	12.03 (<0.0001)	0.68 (0.495)	144.84 (<0.0001)	0.664	0.939	**−4.81 (< 0.001)**

*Note*: A significant departure from isometry for any relationship is highlighted in bold.

^a^ DF = 1.96 for all except for the *m. scapulohumeralis caudalis* when DF = 1.74.

There was a significant interaction term between log body mass and order for the relationship with the mass of *m. pectoralis* (*F*_5,56_ = 2.92, *p* = 0.021) with body mass being a highly significant covariate (*F*_1,56_ = 2289.19, *p* < 0.0001), and order was a significant factor (*F*_5,56_ = 5.34, *p* < 0.001). The model explained most of the variation in muscle mass (*R* ^2^ = 0.977), whilst the phylogenetic signal was low (λ < 0.001).

By contrast, for the mass of the *m. supracoracoideus*, there was no significant interaction between body mass and order (*F*_5,56_ = 1.19, *p* = 0.325). The model was simplified and re‐run and showed highly significant effects of body mass as a covariate and order as a fixed factor (*F*_1,61_ = 1658.09, *p* < 0.0001; *F*_5,61_ = 10.55, *p* < 0.0001, respectively). The model explained most of the variation in the data (*R* ^2^ = 0.966), and the phylogenetic signal was low (λ < 0.001).

Similarly, for the mass of the *m. deltoideus major*, there was no significant interaction between body mass and order (*F*_1,56_ = 1.71, *p* = 0.147), and in the simplified model both body mass and order were highly significant (*F*_1,61_ = 1646.29, *p* < 0.0001; *F*_5,61_ = 16.17, *p* < 0.0001, respectively; *R* ^2^ = 0.966, λ < 0.001).

For the mass of the *m. scapulohumeralis caudalis*, there was also no significant interaction between body mass and order (*F*_4,35_ = 1.08, *p* = 0.382). As for the other muscles, the simplified model showed that both body mass and order were highly significant (*F*_1,39_ = 672.74, *p* < 0.0001; *F*_4,39_ = 9.88, *p* < 0.0001, respectively; *R* ^2^ = 0.948, λ < 0.001).

**FIGURE 3:**
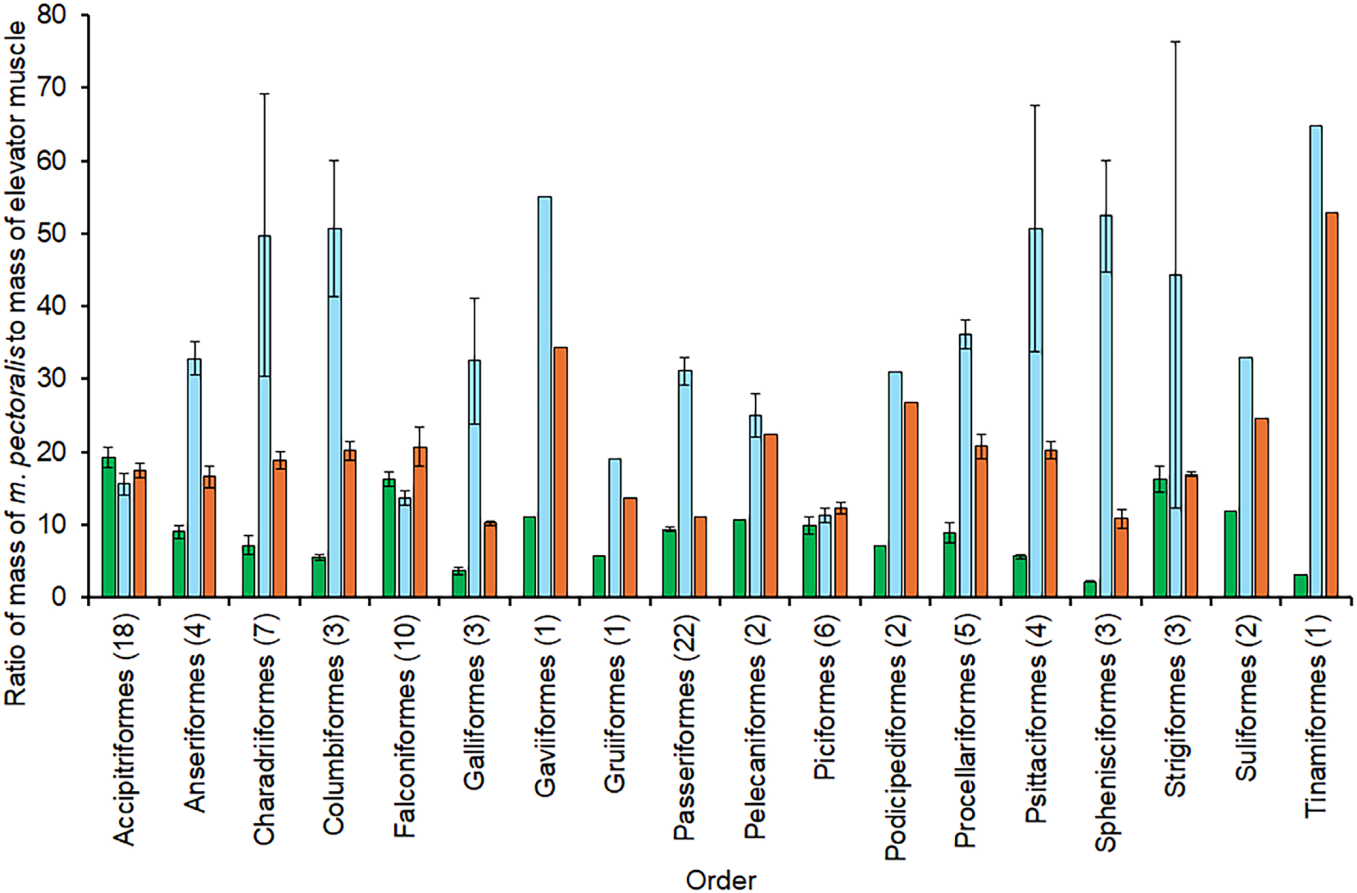
Ratios of the mass of the three elevator muscles relative to mass of the *m. pectoralis*. Values are means, with error bars indicating standard error values where there were at least three representative species in the order. Green bars indicate values for the *m. supracoracoideus*, blue bars indicate values for the *m. deltoideus major*, and orange bars indicate values for the *m. scapulohumeralis caudalis*. Numbers in parentheses indicate the number of species represented in each order.

In general, the mean values for P:DM and P:SHC ratios for the orders represented in the dataset were greater than for the P:SC ratio (Figure 3), that is, relative to the *m. pectoralis*, these muscles were small. In general, the P:DM ratios were greater than the P:SHC ratios, but seemed to be more variable; for instance, for the Accipitriformes, Falconiformes, and Piciformes, the three ratios were comparable in size, but in most other orders, for example, the Strigiformes and Psittaciformes, the P:DM ratio was much larger than the other two ratios (Figure 3). Variance ratio tests showed that the data for the P:DM ratio were significantly more variable than the data for both the P:SC and P:SHC ratios (*F*_96,96_ = 3.74, *p* < 0.001, and *F*_96,44_ = 9.78, *p* < 0.001, respectively). By contrast, the variation ratio test for the comparison between data for P:SC and P:SHC was not significant (*F*_96,44_ = 1.19, *p* > 0.05).

**FIGURE 4:**
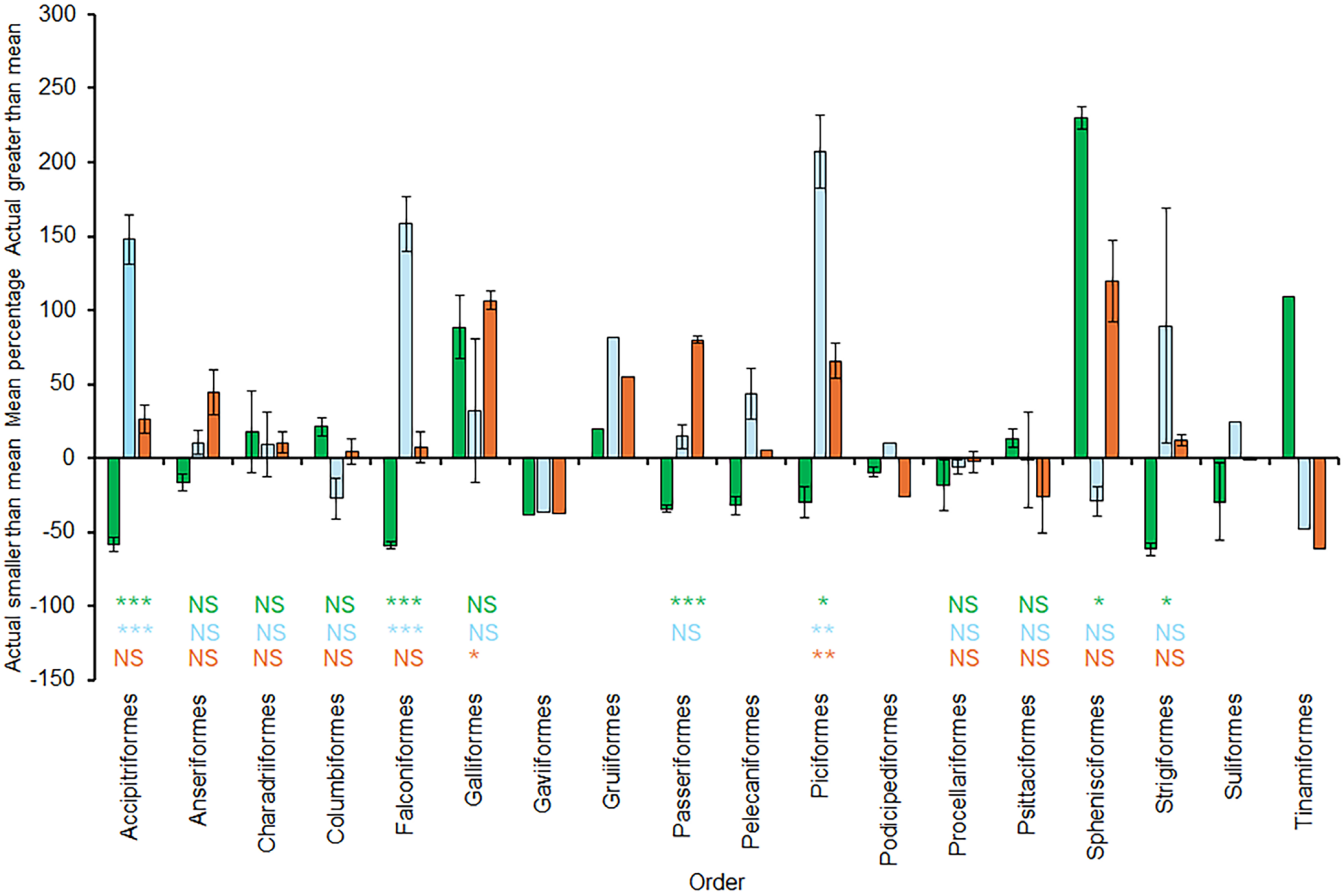
Mean (±SE) for standardised residuals for three elevator muscle mass for orders. Using phylogenetically controlled regression equations, the masses of the *m. supracoracoideus*, *m. deltoideus major*, and *m. scapulohumeralis caudalis* were predicted from the mass of the *m. pectoralis* for each species. The difference between actual and predicted muscle masses was calculated and expressed as a percentage of the actual muscle mass. Asterisks represent significant departures from a mean of zero for each order as determined by one‐way *t*‐tests (NS = non‐significant; *p < 0.05; **p < 0.01; ***p < 0.001; spaces indicate that no test was possible due to sample sizes of 1 or 2); green bars and symbols indicate values and significance for the *m. supracoracoideus*, blue bars and symbols indicate values and significance for the m. deltoideus major, and orange bars and symbols indicate values and significance for the *m. scapulohumeralis caudalis*.

Relative to the predictions based on the mass of *m. pectoralis*, the standardised residuals for each elevator muscle varied among orders (Figure 4). For most orders, there was no significant departure from an expected mean of zero for each muscle. However, for the Piciformes, the *m. supracoracoideus* was significantly lower than predicted from the relationship with the *m. pectoralis* (see Table 2), but the *m. deltoideus major* and *m. scapulohumeralis caudalis* muscles were significantly larger than predicted (Figure 4). For the Accipitriformes and Falconiformes, the *m. supracoracoideus* was significantly lower than predicted from the *m. pectoralis*, but the *m. deltoideus major* muscle was significantly larger than predicted (Figure 4). For the Galliformes, only the *m. scapulohumeralis caudalis* muscle was significantly larger than predicted (Figure 4). Passeriformes and Strigiformes had significantly smaller residuals for the *m. supracoracoideus*, but the value for the Sphenisciformes was significantly larger (Figure 4).

## 4 DISCUSSION

As predicted, all four muscles showed isometric scaling for the relationship between muscle mass and body mass, although for the *m. scapulohumeralis caudalis* exhibited negative allometry in its relationships with the other three muscles. Also, in line with expectations, taxonomic order had a significant effect on the mass of each muscle. Among the three elevator muscles, the P:DM ratio exhibited significantly greater variation than for the P:SC and P:SHC ratios. Notably, the P:DM ratio was significantly higher than the expected ratio of zero for those orders that rely on gliding or soaring flight, for example, the Accipitriformes, Falconiformes, and Piciformes (Figure 4).

Bribiesca‐Contreras et al. (2021) reported that total wing mass scaled isometrically with body mass. Similarly, all of the four muscles studied exhibit isometry with body mass and were significantly influenced by order in terms of the intercepts to relationships. This result aligns with the report of Deeming (2023) for the relationship between body mass and the *m. pectoralis*. However, for the *m. supracoracoideus*, Deeming (2023) reported a slight but significant negative allometry for a much larger dataset, which may reflect the inclusion of species that hover or are burst flyers that have disproportionately large supracoracoideus muscles. Although based on a limited dataset, the results in the present study suggest that the size of these muscles is conserved because of their central role in generating the power required for flight across birds, regardless of body size.

Deeming (2023) also demonstrated that avian order was a significant categorical factor affecting the mass of the *m. pectoralis*. This result is also supported here for the *m. supracoracoideus* and extended to the *m. deltoideus major* and *m. scapulohumeralis caudalis*. This almost certainly reflects differences in flight styles between species, which tend to be more similar within orders. How the differing patterns of muscle sizes affect flight styles is far from clear from this limited dataset, but species that regularly glide or soar (e.g., those of the Accipitriformes or Falconiformes) have, relative to the *m. pectoralis*, significantly smaller masses for the *m. supracoracoideus* and significantly larger masses for the *m. deltoideus major*. By contrast, Galliformes – burst flyers that generate lift through a powerful upstroke – exhibit significantly larger masses for both the *m. supracoracoideus* (Deeming, 2023) and *m. scapulohumeralis caudalis,* whilst the size of the *m. deltoideus major* is as predicted. Interestingly, the Piciformes significantly deviated from the expected for all three elevator muscles. This may reflect the need to use these muscles, especially the *m. deltoideus major*, to stabilise the wing during passive phases of bounding flight when the wings are held horizontally but close to the body (Tobalske, 1996) and, perhaps, represent a functional overlap of its flight modes. Research into the mechanism of flight in birds has concentrated on the actions of the *m. pectoralis* and the *m. supracoracoideus* (e.g., Dial et al., 1997) but this study suggests that there are other sizable elevator muscles, such as the *m. deltoideus major* and *m. scapulohumeralis caudalis*, that need further consideration and investigation in this regard.

It is of interest that the scapular anchor observed in many bird species originates on the scapula but inserts onto the *m. deltoideus major* (Canova et al., 2015; Meyers, 1993; Razmadze et al., 2018). Although this band of connective tissue may play a role as a mechanical stop to limit protraction of the humerus (Meyers, 1993), the muscle to which it inserts could presumably stretch. It will serve as a second point of origin for the *m. deltoideus major* on the scapula, potentially stabilising the muscle during its contraction. As Meyers (1993) suggests, it may be involved in sensing the position of the shoulder joint, thereby indirectly affecting muscle action. Given the importance we attribute to the *m. deltoideus major* in maintaining the humerus during gliding, the functional characteristics of this muscle during gliding and flapping flight would be an interesting area of study.

With the exception of the *m. pectoralis* and *m. supracoracoideus*, the other muscles associated with the pectoral girdle and humerus are light in mass (for example, the *m. pectoralis* is 60–372 times heavier than the *m. latissimus dorsi pars cranialis* in aquatic birds [Bribiesca‐Contreras et al., 2019]) and crucially do not insert on the humerus in a position that would resist any long‐axis rotation of the bone. Often seen as a muscle associated with flexing the shoulder (Proctor & Lynch, 1993) or elevating the humerus (Dial et al., 1991; Hedrick et al., 2004; Beaufrère, 2009; König et al., 2016), the *m. deltoideus major* is considered not to be of sufficient size to raise the wing alone (Proctor & Lynch, 1993). Whilst this muscle has been implicated in rotating the humerus (Bribiesca‐Contreras et al., 2019, 2021; Corvidae et al., 2006) and moving the humerus during flapping flight (Dial, 1992a, 1992b), it would appear that its potential role in gliding flight has received comparatively little attention. Electromyographic studies on the European Starling (*Sturnus vulgaris*) demonstrated that the *m. deltoideus major* is active during both downstroke and upstroke of flapping flight (Dial et al., 1991). By contrast, Meyers (1993) demonstrated that the *m. deltoideus major* was not an active muscle during gliding flight by the American Kestrel, but this species does not normally rely on gliding or soaring. However, these findings might not generalise across taxa with different morphologies or flight styles; ideally, electromyographic studies should be carried out on large birds (e.g., large birds of prey) that regularly glide or soar to determine whether the *m. deltoideus major* is an active muscle during these types of flight.

Variability in the morphology of the *m. deltoideus major* has been recognised by George and Berger (1966), but this study is the first to demonstrate variability in mass ratios among species and orders. The *m. deltoideus major* is relatively large in some, but not all, orders, particularly in those relying on soaring or gliding (e.g., species of the Accipitriformes). An exception is the Procellariiformes, which did not exhibit a particularly large P:DM ratio. This may reflect the small sizes of the species represented in the dataset (≤761 g), which Pennycuick (1982) showed had no sign of a shoulder lock in the *m. pectoralis*. However, it is unclear why these birds, which are reliant on dynamic soaring (Warham, 1996), have relatively small *m. deltoideus major* muscles; perhaps, there is a combination of muscles involved in holding the wing in place, but this will require further investigation.

Our results suggest that in most bird species, the *m. deltoideus major* may play a pivotal role in countering cranially orientated long‐axis rotation of the humerus during contraction of the *m. pectoralis* to hold the wing horizontal. However, it would be good to confirm this suggestion by determining the P:DM ratio for larger species that soar, like albatrosses, cranes (Gruiformes), and storks (Ciconiiformes). Many species within the Accipitriformes also rely on soaring on thermals and do not use flight to catch prey, so they might show an even more developed *m. deltoideus major*. Indeed, the Andean condor (*Vultur gryphus*; Cathartidae) in our dataset had the heaviest *m. deltoideus major* reported: 42 g for an 11.5 kg bird (Hertel et al., 2015), which was a P:DM ratio of 9.8. By contrast, the smaller 1.4 kg turkey vulture (*Cathartes aura*) had a P:DM ratio of 16.3, which was comparable to other Accipitriformes in our dataset. However, the white‐backed vulture (*Gyps africanus*), an Old World vulture of the Accipitridae, had the second largest *m. deltoideus major* reported: 34.5 g for a 5.7 kg individual (Hertel et al., 2015), with a P:DM ratio of 11.9. Further study of the flight musculature of other large soaring New World and Old World vultures would be interesting to determine whether a substantial *m. deltoideus major* mass is typical of these large birds. In addition, if the *m. deltoideus major* is countering long‐axis rotation of the humerus and a change in the orientation of the elbow and ulna, then perhaps it is also deployed in other forms of active flapping where the aerofoil remains relatively flat.

The action of the *m. deltoideus major* may also be affected by the types of muscle fibres (Meyers & McFarland, 2016), which appear to be species‐dependent. For instance, in the double‐crested cormorant, slow fibres formed around 90% of the fibres in the *m. deltoideus minor* (Meyers, 1997), whereas in albatrosses (*Phoebastria* [*Diomedea*] spp.), they account for only 13% of the fibres in the *m. deltoideus major* (Meyers & Stakebake, 2005). Recognising the potential postural role of the *m. deltoideus major* might encourage further investigation into its fibre‐type composition and how this relates to the sustained muscular activity required to maintain a horizontal wing posture during prolonged gliding.

The *m. scapulohumeralis caudalis* inserts on the ventral side of the humerus and is unlikely to be involved with resisting the cranially orientated long‐axis rotation of the humerus, so the finding that its mass deviated significantly from predictions in only a few avian orders was not unexpected. In addition, interspecific variability in its mass was not significantly different from that observed for the *m. supracoracoideus* but was notably lower than that of the *m. deltoideus major*. This suggests that the role of the *m. scapulohumeralis caudalis* is relatively conserved across bird species, regardless of flight style.

Although Procellariiformes are known for their prolonged gliding abilities, species in our dataset did not exhibit particularly large *m. deltoideus major* masses relative to *m. pectoralis*. This may reflect their comparatively small body sizes (<761 g), as Pennycuick (1982) noted that a tendinous ‘shoulder lock’ was only present in larger species such as albatrosses.

Even if the *m. deltoideus major* plays a role in holding the wing horizontally, it remains possible that alternative mechanisms, such as a passive shoulder lock, may also contribute to wing stabilisation in some taxa. However, since larger Procellariiformes were not included in our sample, further study would be required to evaluate the interaction between muscle development and passive structural mechanisms in this group.

In conclusion, as previously suggested, the wing is almost certainly held horizontally during gliding and soaring by contraction of the *m. pectoralis*. However, the *m. deltoideus major* likely plays an important complementary role by countering the long‐axis rotation that such contraction imposes on the humerus. Therefore, the *m. deltoideus major* is largely well developed in species which rely on gliding/soaring, such as the birds of prey, and less developed in species with more active flapping flight, for example, Passeriformes. Further research is needed to explore variability in the size of the *m. deltoideus major* and how this reflects flight style in birds. Studies of the mechanics of flight have often relied on studies of the *m. pectoralis* and *m. supracoracoideus* during active flight, but the results of this study are suggestive of previously unconsidered but substantial roles for other muscles of the pectoral girdle and forelimb during execution of different flight styles. Additionally, the presence of a relatively large *m. deltoideus major* across phylogenetically distant soaring taxa may point towards functional convergence in wing stabilisation. Future studies integrating muscle fibre composition, in vivo muscle activation, and biomechanical modelling will be essential to validate and quantify the contribution of this muscle across flight modes.

### ACKOWLEDGEMENTS

The authors are grateful to the two reviewers for their constructive comments on a previous iteration of this manuscript that helped to improve the final version.

## AUTHOR CONTRIBUTIONS

DCD conceived the ideas and designed the methodology; DCD and MCM collected the data; DCD analysed the data; DCD led the writing of the manuscript. Both authors contributed critically to the drafts and gave final approval for publication.

## CONFLICT OF INTEREST STATEMENT

The authors declare no conflict of interest.

## Supporting information


**Table S1.** Data for body mass and the masses of the *m. pectoralis*, *m. supracoracoideus*, *m. deltoideus major/minor*, and *m. scapulohumeralis caudalis* for 97 species, representing 18 orders. NA indicates datum value is unavailable.

## Data Availability

Data are available in the electronic supplementary materials.
