# Supplementary material for: Holding a wing horizontal: Roles for muscles of the pectoral girdle other than the main two flight muscles
Source: J Anat. 2025 Sep 25;248(5):830–42. doi: 10.1111/joa.70051 (PMC13069140; doi:10.1111/joa.70051)
Supplement: Supplementary file 1 — Table S1. Data for body mass and the masses of the m. pectoralis, m. supracoracoideus, m. deltoideus major/minor, and m. scapulohumeralis caudalis for 97 species, representing 18 orders. NA indicates datum value is unavailable. [file JOA-248-830-s001.docx]

**Holding a wing horizontal involves muscles of the pectoral girdle other than the main two flight muscles**

D. Charles Deeming^1^* & María Clelia Mosto^2^

**Table S1**. Data for body mass and the masses of the *m. pectoralis*, *m. supracoracoideus*, *m. deltoideus major / minor*, and *m. scapulohumeralis caudalis* for 97 species, representing 18 orders. NA indicates datum value is unavailable.

| **Species** | **Body mass (g)** | ***M. pectoralis* mass (g)** | ***M. supracoracoideus* mass (g)** | ***M. deltoideus major / minor* mass (g)** | ***M. scapulohumeralis caudalis* mass (g)** | ***Reference*** |
| --- | --- | --- | --- | --- | --- | --- |
| **Accipitriformes** |  |  |  |  |  |  |
| *Aquila rapax* | 2400 | 168.500 | 6.200 | 11.500 | 8.500 | Hertel et al. (2015) |
| *Accipiter nisus* | 237.5 | 20.330 | 1.163 | 1.198 | 1.267 | Bribiesca-Contreras et al. (2019) |
| *Astur gentilis* | 1137 | 97.500 | 4.100 | 5.580 | 5.590 | This study |
| *Busarellus nigricollis* | 956 | 63.600 | 2.470 | 4.110 | 3.480 | This study |
| *Buteo albigula* | 500 | 42.840 | 2.130 | 2.910 | 2.890 | This study |
| *Buteo buteo* | 875 | 53.917 | 2.613 | 3.489 | 3.349 | Bribiesca-Contreras et al. (2019) |
| *Buteo lineatus* | 600 | 28.000 | 1.800 | 1.600 | 2.100 | Hertel et al. (2015) |
| *Buteogallus coronatus* | 2950 | 152.170 | 8.320 | 14.000 | 11.550 | This study |
| *Cathartes aura* | 1400 | 88.200 | 6.800 | 5.400 | 4.700 | Hertel et al. (2015) |
| *Geranoaetus melanoleucus* | 2751 | 130.820 | 11.760 | 14.570 | NA | This study |
| *Gyps africanus* | 5700 | 412.000 | 17.000 | 34.500 | 14.000 | Hertel et al. (2015) |
| *Haliaeetus albicilla* | 5572 | 276.397 | 18.283 | 19.817 | 17.230 | This study |
| *Milvus milvus* | 1080 | 83.860 | 4.555 | 5.070 | 4.555 | Bribiesca-Contreras et al. (2019) |
| *Pandion haliaetus* | 1568 | 106.167 | 5.250 | 7.457 | 6.740 | This study |
| *Rostrhamus sociabilis* | 446 | 18.520 | 1.030 | 1.220 | 0.920 | This study |
| *Rupornis magnirostris* | 269 | 13.930 | 0.715 | 1.180 | 1.140 | This study |
| *Spizaetus ornatus* | 1421 | 164.000 | 5.200 | 4.240 | 7.120 | This study |
| *Vultur gryphus* | 11500 | 411.000 | 57.000 | 42.000 | 30.000 | Hertel et al. (2015) |
| **Anseriformes** |  |  |  |  |  |  |
| *Anas platyrhynchos* | 1357 | 117.177 | 14.868 | 3.009 | 6.573 | Bribiesca-Contreras et al. (2021) |
| *Mareca strepera* | 870 | 75.527 | 9.368 | 2.259 | 4.057 | Bribiesca-Contreras et al. (2021) |
| *Anser brachyrhynchus* | 2647 | 183.324 | 21.907 | 6.592 | 14.752 | Bribiesca-Contreras et al. (2021) |
| *Chauna torquata* | 4400 | 390.000 | 33.370 | 12.530 | 22.450 | This study |
| **Charadriiformes** |  |  |  |  |  |  |
| *Alle alle* | 97 | 7.442 | 2.235 | 0.410 | 0.336 | Bribiesca-Contreras et al. (2021) |
| *Himantopus himantopus* | 218 | 9.700 | 1.300 | 0.22 | 0.490 | This study |
| *Larus argentatus* | 788 | 47.065 | 4.241 | 1.228 | 2.647 | Bribiesca-Contreras et al. (2021) |
| *Larus fuscus* | 822 | 44.061 | 4.123 | 1.194 | 2.300 | Bribiesca-Contreras et al. (2021) |
| *Rissa tridactyla* | 172 | 5.311 | 0.953 | 0.194 | 0.412 | Bribiesca-Contreras et al. (2021) |
| *Uria aalge* | 595 | 45.830 | 16.684 | 0.280 | 2.098 | Bribiesca-Contreras et al. (2021) |
| *Vanellus chilensis* | 327 | 19.880 | 2.120 | 1.05 | 1.08 | This study |
| **Columbiformes** |  |  |  |  |  |  |
| *Patagioenas maculosa* | 347 | 36.150 | 6.720 | 1.030 | 2.020 | This study |
| *Patagioenas picazuro* | 279 | 47.190 | 7.530 | 0.700 | 2.320 | This study |
| *Zenaida auriculata* | 136 | 15.800 | 3.145 | 0.320 | 0.705 | This study |
| **Pelecaniformes** |  |  |  |  |  |  |
| *Ardea cocoi* | 3200 | 120.900 | 12.420 | 5.480 | 6.675 | This study |
| *Ardea alba* | 935 | 94.810 | 8.240 | 3.390 | 3.540 | This study |
| **Falconiformes** |  |  |  |  |  |  |
| *Caracara plancus* | 1358 | 71.970 | 5.260 | 7.250 | 4.530 | Picasso & Mosto (2018) |
| *Falco columbarius* | 190.5 | 11.795 | 0.782 | 0.595 | 0.636 | Bribiesca-Contreras et al. (2019) |
| *Falco femoralis* | 311 | 20.585 | 1.080 | 1.300 | 1.060 | Mosto et al. (2022) |
| *Falco peregrinus* | 757.2 | 94.010 | 5.590 | 7.590 | 2.240 | Bribiesca-Contreras et al. (2019), Mosto et al. (2022) |
| *Falco sparverius* | 103.6 | 5.397 | 0.355 | 0.377 | 0.297 | Mosto et al. (2022) |
| *Falco subbuteo* | 144 | 4.100 | 0.350 | 0.260 | 0.260 | Mosto et al. (2022) |
| *Falco tinnunculus* | 176.9 | 10.500 | 0.570 | 0.730 | 0.470 | Bribiesca-Contreras et al. (2019), Mosto et al. (2022) |
| *Daptrius chimachima* | 315.5 | 15.260 | 0.680 | 1.260 | 0.640 | Picasso & Mosto (2018) |
| *Daptrius chimango* | 302.5 | 15.990 | 1.080 | 1.290 | 0.785 | Picasso & Mosto (2018) |
| *Micrastur ruficollis* | 196 | 8.710 | 0.580 | 0.890 | 0.800 | This study |
| **Galliformes** |  |  |  |  |  |  |
| *Alectoris chukar* | 541 | 39.476 | 12.893 | 0.9335 | 3.717 | Heers et al. 2018 |
| *Chrysolophus pictus* | 710 | 28.830 | 8.495 | 0.719 | 2.771 | Yang et al. (2015) |
| *Penelope obscura* | 1770 | 39.730 | 8.630 | 2.630 | 4.080 | This study |
| **Gaviiformes** |  |  |  |  |  |  |
| *Gavia stellata* | 1255 | 47.759 | 4.283 | 0.869 | 1.387 | Bribiesca-Contreras et al. (2021) |
| **Gruiiformes** |  |  |  |  |  |  |
| *Aramides ypecaha* | 737 | 38.930 | 6.850 | 2.045 | 2.835 | This study |
| **Tinamiformes** |  |  |  |  |  |  |
| *Nothura maculosa* | 260 | 20.070 | 6.410 | 0.310 | 0.380 | This study |
| **Podicipediformes** |  |  |  |  |  |  |
| *Podiceps major* | 1646 | 26.910 | 3.520 | 0.990 | 0.950 | This study |
| *Rollandia rolland* | 424 | 5.580 | 0.860 | 0.160 | 0.220 | This study |
| **Procellariformes** |  |  |  |  |  |  |
| *Calonectris diomedea* | 761 | 33.474 | 2.609 | 0.827 | 1.528 | Bribiesca-Contreras et al. (2021) |
| *Fulmarus glacialis* | 535 | 15.303 | 1.800 | 0.481 | 0.602 | Bribiesca-Contreras et al. (2021) |
| *Pachyptila vittata* | 158.5 | 7.255 | 0.744 | 0.230 | 0.452 | Bribiesca-Contreras et al. (2021) |
| *Halobaena caerulea* | 202 | 5.133 | 0.580 | 0.127 | 0.293 | This study |
| *Puffinus puffinus* | 390 | 13.050 | 3.040 | 0.360 | 0.570 | Bribiesca-Contreras et al. (2021) |
| **Psittaciformes** |  |  |  |  |  |  |
| *Amazona vinacea* | 254 | 18.000 | 3.300 | 0.810 | 1.000 | This study |
| *Nymphicus hollandicus* | 87.28 | 9.100 | 1.410 | 0.430 | NA | Hedrick et al. (2004) |
| *Primolius auricollis* | 245 | 13.000 | 2.260 | 0.150 | 0.630 | This study |
| *Psittacus erithacus* | 333 | 11.346 | 2.247 | 0.157 | 0.513 | Razmadze et al. (2018) |
| **Sphenisciformes** |  |  |  |  |  |  |
| *Eudyptes moseleyi* | 2500 | 164.648 | 70.563 | 3.270 | 13.076 | Bribiesca-Contreras et al. (2021) |
| *Spheniscus demersus* | 2900 | 93.763 | 44.025 | 2.329 | 11.430 | Bribiesca-Contreras et al. (2021) |
| *Spheniscus humboldti* | 4200 | 156.030 | 72.527 | 2.329 | 13.348 | Bribiesca-Contreras et al. (2021) |
| **Strigiformes** |  |  |  |  |  |  |
| *Glaucidium brasilianum* | 77.2 | 4.160 | 0.210 | 0.320 | 0.240 | This study |
| *Glaucidium nana* | 81.2 | 3.420 | 0.250 | 0.300 | 0.210 | This study |
| *Tyto furcata* | 330 | 19.060 | 1.243 | 0.176 | 1.113 | This study |
| **Suliformes** |  |  |  |  |  |  |
| *Morus bassanus* | 2923.79 | 194.574 | 11.696 | 4.268 | 6.104 | Bribiesca-Contreras et al. (2021) |
| *Gulosus aristotelis* | 1034.000 | 43.519 | 6.158 | 2.153 | 2.523 | Bribiesca-Contreras et al. (2021) |
| **Piciformes** |  |  |  |  |  |  |
| *Colaptes melanochloros* | 140.000 | 10.480 | 0.780 | 0.98 | 0.690 | This study |
| *Colaptes pitius* | 159.000 | 10.540 | 0.840 | 1 | 0.850 | This study |
| *Pteroglossus castanotis* | 273.000 | 14.110 | 1.390 | 0.935 | 1.150 | This study |
| *Ramphastus sulfuratus* | 433.000 | 6.980 | 1.330 | 0.86 | 0.770 | This study |
| *Ramphastos toco* | 618.000 | 37.000 | 3.970 | 3.33 | 3.340 | This study |
| *Selenidera maculirostris* | 164.000 | 12.360 | 1.370 | 1 | 0.900 | This study |
| **Passeriformes** |  |  |  |  |  |  |
| *Acrocephalus melanopogon* | 11.146 | 1.0937 | 0.196 | 0.048 | NA | Calmaestra & Moreno 2005 |
| *Acrocephalus scirpaceus* | 11.867 | 1.3683 | 0.184 | 0.042 | NA | Calmaestra & Moreno 2005 |
| *Lanius excubitor* | 60.833 | 8.6814 | 0.921 | 0.283 | NA | Calmaestra & Moreno 2005 |
| *Lanius senator* | 32.273 | 5.4492 | 0.501 | 0.140 | NA | Calmaestra & Moreno 2005 |
| *Motacilla cinerea* | 16.687 | 3.5987 | 0.400 | 0.104 | NA | Calmaestra & Moreno 2005 |
| *Motacilla flava* | 17.181 | 3.8698 | 0.337 | 0.080 | NA | Calmaestra & Moreno 2005 |
| *Oenanthe leucura* | 36.592 | 4.8245 | 0.524 | 0.238 | NA | Calmaestra & Moreno 2005 |
| *Oenanthe oenanthe* | 22.788 | 4.9536 | 0.486 | 0.158 | NA | Calmaestra & Moreno 2005 |
| *Phoenicurus ochruros* | 15.954 | 3.2236 | 0.343 | 0.136 | NA | Calmaestra & Moreno 2005 |
| *Phoenicurus phoenicurus* | 15.219 | 3.388 | 0.303 | 0.105 | NA | Calmaestra & Moreno 2005 |
| *Phylloscopus collybita* | 7.440 | 1.321 | 0.161 | 0.042 | NA | Calmaestra & Moreno 2005 |
| *Phylloscopus trochilus* | 8.912 | 1.7954 | 0.213 | 0.055 | NA | Calmaestra & Moreno 2005 |
| *Pyroderus scutatus* | 357.00 | 25.62 | 2.110 | 1.400 | 2.200 | This study |
| *Saxicola rubetra* | 16.839 | 3.1817 | 0.325 | 0.100 | NA | Calmaestra & Moreno 2005 |
| *Saxicola torquatus* | 15.682 | 2.4951 | 0.273 | 0.100 | NA | Calmaestra & Moreno 2005 |
| *Sturnus unicolor* | 84.495 | 14.887 | 1.578 | 0.351 | NA | Calmaestra & Moreno 2005 |
| *Sturnus vulgaris* | 76.632 | 17.469 | 1.729 | 0.351 | NA | Calmaestra & Moreno 2005 |
| *Curruca communis* | 16.202 | 2.4388 | 0.288 | 0.078 | NA | Calmaestra & Moreno 2005 |
| *Curruca melanocephala* | 11.795 | 1.6745 | 0.217 | 0.063 | NA | Calmaestra & Moreno 2005 |
| *Turdus iliacus* | 60.633 | 11.188 | 1.106 | 0.300 | NA | Calmaestra & Moreno 2005 |
| *Turdus merula* | 84.117 | 12.505 | 1.425 | 0.441 | NA | Calmaestra & Moreno 2005 |
| *Turdus rufiventris* | 72.200 | 6.01 | 0.647 | 0.410 | 0.580 | This study |

**References**

Bribiesca‑Contreras, F., Parslew, B. & Sellers, W.I. (2019). A Quantitative and Comparative Analysis of the Muscle Architecture of the Forelimb Myology of Diurnal Birds of Prey (Order Accipitriformes and Falconiformes). *The Anatomical Record*, 302, 1808–1823.

Bribiesca‑Contreras, F., Parslew, B. & Sellers, W.I. (2021). Functional morphology of the forelimb musculature reflects flight and foraging styles in aquatic birds. *Journal of Ornithology*, 162:779–793.

Calmaestra, R.G. & Moreno, E. (2005). Forelimb muscles and migration: finding ecomorphological patterns using a phylogenetically-based method. *Ardeola* 52, 253-268.

Hedrick, T.L., Usherwood, J.R., & Andrew A. Biewener, A.A. (2004). Wing inertia and whole-body acceleration: an analysis of instantaneous aerodynamic force production in cockatiels (*Nymphicus hollandicus*) flying across a range of speeds. *Journal of Experimental Biology*, 207, 1689-1702.

Heers, A.M., Rankin, J.W., & Hutchinson, J.R. (2018). Building a bird: musculoskeletal modeling and simulation of wing-assisted incline running during avian ontogeny. *Frontiers in Bioengineering and Biotechnology*, 6:140.

Hertel, F., Maldonado, J. E., & Sustaita, D. (2015). Wing and hindlimb myology of vultures and raptors (Accipitriformes) in relation to locomotion and foraging. *Acta Zoologica*, **96,** 283–295.

Mosto, M. C., Picasso, M. B. J., Montes, M. M., Tudisca, A. M., & Krone, O. (2022). Flight muscles in falcons (Falconiformes, Falconinae): A quantitative approach. *The Anatomical Record*, 305, 1287–1293.

Picasso, M.B.J. & Mosto, M.C. (2018). Wing myology of Caracaras (Aves, Falconiformes): muscular features associated with flight behavior. *Vertebrate Zoology*, 68, 177-190.

Razmadze, D. Panyutina, A. A., & Zelenkov, N.V. (2018). Anatomy of the forelimb musculature and ligaments of *Psittacus erithacus* (Aves: Psittaciformes). *Journal of Anatomy*, 233, 496—530.

Yang, Y., Wang, H., & Zhang, Z. (2015) Muscle architecture of the forelimb of the Golden Pheasant (Chrysolophus pictus) (Aves: Phasianidae) and its implications for functional capacity in flight. *Avian Research*, 6, 3.
